# Severity of Inflammation Is Associated with Food Intake in Hospitalized Geriatric Patients—A Merged Data Analysis

**DOI:** 10.3390/nu15143079

**Published:** 2023-07-08

**Authors:** Maryam Pourhassan, Tommy Cederholm, Lorenzo M. Donini, Eleonora Poggiogalle, Ursula Schwab, Rikke Lundsgaard Nielsen, Aino Leegaard Andersen, Sylwia Małgorzewicz, Dorothee Volkert, Rainer Wirth

**Affiliations:** 1 Department of Geriatric Medicine, Marien Hospital Herne, Ruhr-Universität Bochum, 44625 Herne, Germany; rainer.wirth@elisabethgruppe.de; 2 Department of Public Health and Caring Sciences, Clinical Nutrition and Metabolism, Uppsala University, 75122 Uppsala, Sweden; tommy.cederholm@pubcare.uu.se; 3 Theme Inflammation & Aging, Karolinska University Hospital, 14186 Stockholm, Sweden; 4 Department of Experimental Medicine, University of Rome “Sapienza”, 00185 Rome, Italy; lorenzomaria.donini@uniroma1.it (L.M.D.); eleonora.poggiogalle@uniroma1.it (E.P.); 5 Institute of Public Health and Clinical Nutrition, School of Medicine, University of Eastern Finland, 70210 Kuopio, Finland; ursula.schwab@uef.fi; 6 Department of Clinical Research, ACUTE-CAG, Copenhagen University Hospital, 2650 Hvidovre, Denmark; rikke.lundsgaard.nielsen@regionh.dk (R.L.N.); aino.leegaard.andersen@regionh.dk (A.L.A.); 7 Department of Clinical Nutrition, Medical University of Gdańsk, 80-210 Gdańsk, Poland; sylwia@tetra.pl; 8 Institute for Biomedicine of Aging, Friedrich-Alexander-Universität Erlangen-Nürnberg, 90403 Nuremberg, Germany; dorothee.volkert@fau.de

**Keywords:** food intake, inflammation, C-reactive protein, older persons, GLIM criteria

## Abstract

The extent to which inflammation impacts food intake remains unclear, serving as a key risk factor for malnutrition as defined by the Global Leadership Initiative on Malnutrition (GLIM). To address this, we analyzed a large, merged dataset of geriatric hospitalized patients across Europe. The study included 1650 consecutive patients aged ≥65 year from Germany, Italy, Finland, Denmark, and Poland. Nutritional intake was assessed using the first item of the Mini Nutritional Assessment Short Form; C-reactive protein (CRP) levels were measured using standard procedures. In total (age 79.6 ± 7.4 year, 1047 females), 23% exhibited moderate to severe inflammation, and 12% showed severe inflammation; 35% showed moderate reductions in food intake, and 28% were considered malnourished. Median CRP levels differed significantly between patients with severe, moderate, and no decrease in food intake. Among patients with a CRP level of 3.0–4.99 mg/dL, 19% experienced a severe decrease in food intake, while 66% experienced moderate to severe decreases. Regression analysis revealed that inflammation was the most prominent risk factor for low food intake and malnutrition, surpassing other factors such as age, gender, infection, and comorbidity. A CRP level of ≥3.0 mg/dL is associated with reduced food intake during last 3 months in two thirds of hospitalized geriatric patients and therefore indicative for a high risk of malnutrition.

## 1. Introduction

Malnutrition represents a crucial health concern in older patients due to its frequent co-occurrence with other diseases and its association with severe outcomes, such as a reductions in quality of life, prolonged hospital stays, and increased rates of mortality and morbidity [1,2]. Prior publications in the literature have demonstrated that the prevalence of malnutrition among older patients could potentially be as high as 50% [3]. However, it is critical to bear in mind that these estimates are subject to significant variation based on several key factors. The demographic characteristics of the studied population, the healthcare setting in which evaluation occurs, and the specific method/or tools used for malnutrition assessment all contribute to this variation. A systematic review and meta-analysis utilizing 22 validated malnutrition screening tools for older adults ≥ 65 years disclosed an intriguing range in malnutrition risk, from a low of 8.5% among community-dwelling elderly to as high as 28% among older hospitalized patients [4].

Various mechanisms contribute to the development of malnutrition, with a key factor being the disparity between the energy consumed and the energy required by the body. This energy imbalance, which is mainly characterized by insufficient energy intake, is a common occurrence in older individuals [5]. However, the mechanisms underlying this imbalance are multifaceted and include physiological changes such as a decrease in basal metabolic rate and impaired absorption of nutrients due to age-related changes in the gastrointestinal tract, age-related alterations in hormone levels, and acute or chronic diseases (e.g., infections, surgery, cardiovascular disease, and diabetes) [2,6,7,8]. Additional contributing factors encompass poor dental health, difficulties in chewing and swallowing, pharmacological treatment side effects, cognitive impairments, and social factors such as isolation, loneliness, and depression, which can negatively affect appetite and energy balance, accelerating a decline in food consumption among this patient group [2]. Therefore, the development of malnutrition in older individuals is multifactorial and complex; however, its etiology is not entirely understood. 

Associated with many acute and chronic diseases, inflammation appears to have a significant influence on the underlying pathophysiological processes associated with malnutrition. Beyond the molecular and cellular catabolic effects that lead to fat and muscle tissue breakdown, inflammation can result in anorexia, which is characterized by a loss of appetite and low nutritional intake [9,10,11]. Acute illnesses may trigger inflammatory responses within body tissues that can potentially disrupt typical dietary behaviors in patients. This is particularly noteworthy in the context of older patients, where such disruptions may lead to adverse effects on their health-related quality of life [9,12]. Indeed, the inflammatory processes initiated by acute illnesses can stimulate physiological changes that alter appetite and feeding behavior, thereby influencing nutritional intake and ultimately affecting patient wellbeing [11,13,14]. Due to its overall catabolic role, inflammation was included as an etiologic criterion in the global consensus on the diagnostic criteria of malnutrition, which was published by the Global Leadership Initiative on Malnutrition (GLIM) [15] in 2019. The criteria for diagnosing malnutrition include three phenotype aspects: unintentional weight loss, a below-normal body mass index, and/or a decline in muscle mass, as well as two etiologic aspects: impaired food intake/assimilation and the presence of inflammation or a high burden of disease [15]. For a malnutrition diagnosis to be confirmed, at least one abnormality must be present in both the phenotypic and etiologic criteria.

C-reactive protein (CRP), identified as a non-specific biological marker of systemic inflammation, exhibits increased levels during periods of both acute and chronic inflammation, as supported by results reported in the literature [16,17]. This indicator is a responsive measure of the body’s inflammatory state and is subject to fluctuations in response to changes in the body’s overall health. Higher levels of CRP have been linked with changes in dietary behaviors, specifically a decrease in food intake [18]. Under normal health conditions, serum CRP levels remain below 0.5 mg/dL [19,20]. However, in the context of older hospitalized patients, increased serum CRP levels have been commonly associated with reduced food intake [11]. 

Currently, the degree of inflammation that significantly influences dietary intake and nutritional status, and thus poses a substantial risk factor for malnutrition, remains unclear. In our previous single-center study conducted in Germany with a cohort of 377 geriatric hospital patients [21], we addressed this question. Our results suggested that patients with a CRP above 3.0 mg/dL demonstrate significantly less food intake than those without inflammation. To substantiate and augment these initial findings, we sought to replicate the investigation in a larger cohort and diversify our sample population by incorporating multiple study centers. To achieve this, we merged suitable datasets on older hospitalized patients from previous studies across Europe to perform this analysis. By merging these data, we aimed to conduct a more comprehensive analysis of the relationship between inflammation and food intake and thus further elucidate the role of inflammation in the etiology of malnutrition among hospitalized geriatric patients.

## 2. Materials and Methods

This multicenter study is a joint analysis of six cross-sectional datasets from Germany [21,22,23], Italy [24,25], Denmark [26], Finland, and Poland (not yet published). Members of the special interest group “Geriatrics” of the European Society of Clinical Nutrition and Metabolism (ESPEN) planned the study and provided the datasets. The study population comprised 1901 consecutive hospitalized older participants, of which 251 subjects had to be excluded from the analyses due to missing values of either serum CRP or Mini Nutritional Assessment Short Form (MNA-SF). Finally, 1650 participants (677 patients from two centers in Germany, 488 from Italy, 228 from Finland, 193 from Denmark, and 64 from Poland) aged between 65 and 100 years (1047 females) were included in the analysis. Patient eligibility criteria were age ≥ 65 years and the ability to provide written informed consent. Further in- and exclusion criteria of the aforementioned studies are described in the respective original publications [21,22,23,24,25,26]. Data for this analysis was collected during the initial days following hospital admission. Every participant involved in the study provided their written informed consent.

### 2.1. Assessment of Nutritional Status and Food Intake

In all centers, the MNA-SF was used to evaluate the nutritional status of the patients. The MNA-SF considers changes in food consumption and weight over the previous three months, mobility, the presence of psychological stress or acute illness, neuropsychological issues, and BMI or, alternatively, calf circumference measurements [27]. The resulting scores categorized patients into three groups: those with normal nutritional status (12–14 points), those at risk of malnutrition (8–11 points), and those classified as malnourished (0–7 points). Further, nutritional intake was evaluated using the food intake item of MNA-SF, where participants are classified as demonstrating no, moderate, or a severe decrease in food intake over the last three months due to a loss of appetite, digestive complications, or difficulties in chewing or swallowing.

### 2.2. Geriatric Assessment 

The capacity for self-care activities was quantified using the Barthel scale [28], a comprehensive measure for evaluating one’s ability to perform daily activities. The scoring for the German version of the Barthel scale ranges from 0 to 100 points, with a score of 100 indicating complete independence in performing everyday tasks, thus reflecting a high level of physical functioning. We used the FRAIL scale [29] as an instrument to diagnose frailty. The FRAIL scale provides a score between 0 and 5, where 0 signifies no frailty, scores of 1–2 are indicative of a pre-frail state, and a score of 3–5 suggests the presence of frailty. Further, the risk of sarcopenia was assessed using the SARC-F questionnaire [30]. This tool encompasses a total score of 10, with individuals scoring 4 or above being categorized as having a probable risk of sarcopenia. To evaluate the cognitive function of our participants, we evaluated patients according to the Montreal Cognitive Assessment (MoCA) [31]. The MoCA has a maximum score of 30, with a score of 26 or higher generally considered as normal cognitive function. Moreover, to assess the prevalence and intensity of depressive symptoms, we used the Depression in Old Age Scale [32]. According to this scale, scores of 0–2 are indicative of an absence of depression, a score of 3 is suggestive of possible depression, and scores within the 4–10 range are indicative of probable depression. Lastly, to establish the extent of medical comorbidities, the Charlson Comorbidity Index [33] was utilized. This widely accepted tool provides a composite score that takes into account the number and severity of comorbid conditions, thereby helping to determine the overall health status of the individual.

### 2.3. Assessment of C-Reactive Protein

Standard hospital procedures were adhered to for the analysis of the C-reactive protein (CRP). Serum CRP levels ranging from 0.0 to 0.49 mg/dL were categorized as no inflammation (or normal values). Mild inflammation was considered at levels between 0.5 and 3.0 mg/dL, while levels of 3.0 mg/dL and above indicated moderate to severe inflammation [19,20]. For the purposes of assessing the proportions of patients with varying degrees of decreased food intake at different CRP levels, the CRP levels were divided into six distinct groups: 0.0–0.99 mg/dL, 1.0–1.99 mg/dL, 2.0–2.99 mg/dL, 3.0–4.99 mg/dL, 5.0–9.99 mg/dL, and ≥10.0 mg/dL.

### 2.4. Data Analysis 

Data analysis was performed using SPSS software (SPSS Statistics for Windows, IBM Corp, Version 27.0, Armonk, NY, USA). We expressed continuous variables either as mean values accompanied by standard deviations (SD) in cases where the data followed a normal distribution or as median values alongside interquartile ranges (IQR) for datasets that deviated from a normal distribution pattern. Categorical variables, on the other hand, were represented in terms of absolute counts and relative frequencies and expressed as percentages. To identify differences in CRP values in relation to various levels of MNA-SF and food intake, we performed the Kruskal–Wallis test (supplemented with pairwise comparisons). Additionally, we assessed variations in the proportion of patients exhibiting moderate to severe decreases in food intake across different CRP categories using the one-way ANOVA Tukey test or the Chi-square test as appropriate. A multiple regression analysis was utilized to explore the influence of risk factors such as inflammation, Charlson Comorbidity Index, age, and gender (as independent variables) on moderate-to-severe and severe food intake reduction, as well as on malnutrition (as dependent variables). Moreover, the impact of aforementioned risk factors, along with infection, on severe food intake reduction was tested within a subgroup of patients where infection data were available. The threshold for statistical significance was conventionally set at *p* < 0.05.

## 3. Results

### 3.1. Baseline Characteristics

The baseline attributes of the study cohort are outlined in Table 1. The study population, with a mean age of 79.6 ± 7.4 years, consisted of 63% females. Among these individuals, it is worthy to note that 23% (or 376 patients) exhibited a CRP level exceeding 3.0 mg/dL. The Body Mass Index (BMI) values of the study group spanned a considerable spectrum, ranging from as low as 10.7 to as high as 63.3 kg/m^2^, with no significant differences being observed between sexes (*p* = 0.755). According to MNA-SF, 41% of patients were at risk of malnutrition, while 28% were already malnourished and 31% had normal nutritional status. It was also observed that 12% and 35% of the population had experienced a severe and moderate decrease in food intake. Interestingly, nearly half of these individuals were found to have moderate to severe inflammation. 

The primary reasons leading to hospitalization within this group included infections, fall-related injuries and fractures, osteoarthritis, post-stroke care requirements, and cardiovascular and neurodegenerative disorders. As indicated by the geriatric assessment data, which were only available for a subset of the study participants, the study population showed the typical degrees of multimorbidity, cognitive impairment, and frailty for geriatric hospital patients (median and interquartile range: Charlson Comorbidity Index: 2, 1–3 (*n* = 1302); Montreal Cognitive Assessment: 18, 14–22 (*n* = 345); Depression in Old Age Scale: 3, 1–5 (*n* = 353); Barthel scale: 50, 40–70 (*n* = 673); FRAIL-Scale: 4, 3–5 (*n* = 398); and SARC-F: 6, 5–8 (*n* = 397)). Further, infection data were available for 650 patients, of which 25% (*n* = 165) had an infectious disease (mostly pneumonia and urinary tract infection) at the time they were administered to hospital.

### 3.2. Comparison of CRP Concentrations across the MNA-SF and Food Intake Levels 

**Figure 1:**
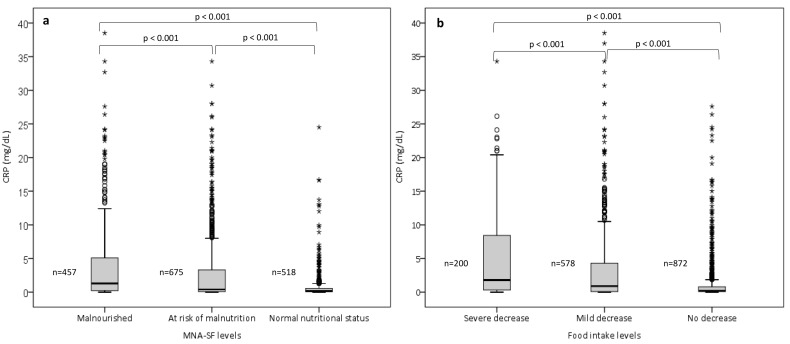
Comparison of CRP values across the (**a**) MNA-SF levels and (**b**) food intake levels in total population (*n* = 1650). MNA-SF, Mini Nutritional Assessment Short Form; CRP, C-reactive protein. Nutritional intake was evaluated using the food intake item of MNA-SF.

A substantial variation was observed in the median CRP concentrations when stratified by the categories of the MNA-SF (Figure 1a). Patients classified as malnourished exhibited a significantly elevated median CRP value (3.9 ± 5.9 mg/dL) in contrast to those at risk of malnutrition (2.9 ± 5.4 mg/dL) or having a normal nutritional status (0.8 ± 2.3 mg/dL, *p* < 0.001). Similarly, noticeable differences in median CRP concentrations were observed when these were grouped according to varying levels of food intake (Figure 1b). The median CRP values demonstrated a significant difference among groups with a severe (5.2 ± 6.7 mg/dL), moderate (3.5 ± 5.9 mg/dL), and no decrease (1.3 ± 3.3 mg/dL) in food intake (*p* < 0.001, Figure 1b). 

### 3.3. Relationship between Inflammation and Food Intake 

**Figure 2:**
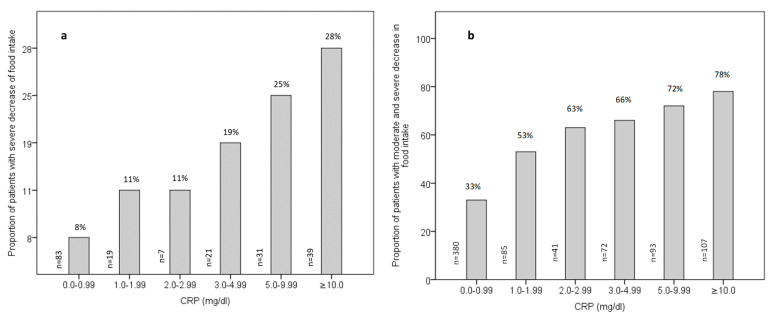
Percentage of patients with (**a**) severe decrease in food intake (*n* = 200) and (**b**) moderate and severe decrease in food intake (*n* = 778) across the categories of CRP levels. CRP, C-reactive protein. Nutritional intake was evaluated using the food intake item of MNA-SF.

Figure 2a,b show the percentage of patients experiencing severe and moderate to severe reductions in food intake across varying levels of CRP, respectively. The proportion of patients undergoing a severe decrease in food intake remained statistically similar at CRP levels of 0.0–0.99 mg/dL, 1.0–1.99 mg/dL, and 2.0–2.99 mg/dL. However, it exhibited a substantial increase at a CRP level of 3.0 mg/dL and above (from 8% at CRP levels 0.0–0.99 mg/dL to 19% at CRP levels 3.0–4.99 mg/dL; *p* = 0.006, Figure 2a). Additionally, when CRP levels were divided into two groups (<3 and ≥3 mg/dL), the percentage of a severe reduction in food intake was significantly lower in patients with CRP levels < 3 mg/dL compared to those with CRP levels ≥ 3 mg/dL (9% vs. 25%; *p* < 0.001).

Comparable findings were observed for individuals exhibiting moderate and severe reductions in food intake. The proportion of patients with moderate and severe reductions in food intake were two times higher at CRP levels between 3.0 and 4.99 mg/dL (66%) compared to 33% at CRP levels between 0.0 and 0.99 mg/dL (*p* < 0.001, Figure 2b). Additionally, the data revealed a markedly larger increase in the proportion of patients experiencing moderate to severe reductions in food intake at a CRP level of ≥3mg/dL (72%) relative to a CRP level of <3 mg/dL (40%, *p* < 0.001).

To test the independent effect of factors such as inflammation, age, gender, and the Charlson Comorbidity Index on severe decrease in food intake (as dependent variable), we performed a series of multiple regression analyses (Table 2). In our first assessment, which included the total population, inflammation (*p* < 0.000) and being male (*p* = 0.007) were identified as the key independent determinants of a severe decline in food intake, accounting for 5.7% and 0.7% of the variance, respectively. On the contrary, other factors such as age (*p* = 0.262) and Charlson Comorbidity Index (*p* = 0.511) were devoid of any significant link to a marked decline in food intake. In our second analysis, which considered both moderate and severe food intake reductions as the dependent variable, we observed a further rise in the explained variance (11.1%). Here, inflammation, age, and gender explained 8.6%, 1.2%, and 1.3% of the variance, respectively. Using malnutrition (yes, no) as the dependent variable in the regression analysis, CRP, age, Charlson Comorbidity Index, and male gender were independent predictors of malnutrition, with CRP explaining of the variance 5.7%, age explaining 2.9% of the variance, and CCI and male gender each explaining 0.4% of the variance (Table 2).

In the following step, we evaluated the effects of the previously mentioned risk factors and infection on severe food intake reduction within a subset of patients for whom infection data were available (*n* = 650). Inflammation (*p* < 0.000) remained the most significant independent risk factor for a severe decrease in food intake, accounting for 4.5% of the variance, while being male (*p* = 0.024) had a more modest impact, explaining just 1.0% of the variance. Other factors, namely age (*p* = 0.753), Charlson Comorbidity Index (*p* = 0.124), and infection (*p* = 0.963), failed to demonstrate a significant relationship with diminished food intake. 

## 4. Discussion

The findings of the present study reveal a pronounced inverse relationship between inflammation and food intake among older hospitalized patients across five European nations. More specifically, there was a significant increase in the proportion of patients exhibiting either a severe or moderate to severe reduction in food intake in the subset with CRP levels equal to or greater than 3 mg/dL in comparison to those with levels below 3 mg/dL. Numerous observational studies have demonstrated the robust association between inflammation and various physiological changes, including loss of appetite [9,13,14,34,35,36,37], reduced food intake [11] and weight loss and low BMI [10,38], across diverse patient groups. This consistent pattern suggests a systemic impact of inflammation in the body’s metabolic processes and dietary behaviors.

Our study, in concert with prior research [21], has elucidated a dose–effect relationship between inflammation and changes in appetite and/or food intake [13,34]. In a cross-sectional study involving older hospitalized patients, Sieske and colleagues reported that nearly 70% of patients with CRP levels greater than 3.0 mg/dL consumed food at a rate that fell below 75% of their dietary requirements [9]. Moreover, results from a randomized, double-blind nutritional intervention study conducted on a cohort of 455 hospitalized older patients further confirmed this association [39]. The study revealed that elderly patients with elevated CRP levels (CRP > 1 mg/dL) demonstrated significantly lower energy intake [39]. This relationship is further reinforced by interventional animal studies demonstrating decreased food intake following the administration of pro-inflammatory cytokines such as IL18 [40]. Collectively, these findings provide compelling evidence that indicates a causative role for inflammation in modulating food intake. However, the mechanistic details remain unclear, and it is not yet certain which molecular signals of inflammation are most important for appetite regulation [14]. 

In older adults, inflammation and malnutrition often present concomitantly. This prominent association has underscored the role of inflammation as a significant risk factor for malnutrition. Consequently, inflammation has been included into the internationally recognized diagnostic criteria for malnutrition [15], a decision further supported by the data from our current study. The original framework formulated by the GLIM identified inflammation as an etiological factor for malnutrition, with particular emphasis being placed on prolonged catabolism and anorexia, which is often observed during acute and chronic disease states [15]. However, the GLIM paper did not provide guidance on relevant cut-off values for inflammation. Upon the release of the GLIM construct, several researchers began applying relatively mild inflammation thresholds, independent of chronic disease, to fulfill the inflammation criterion of GLIM.

As an illustration, a cohort study involving 411 community-dwelling older participants (with a mean age of 72.3 ± 6.1 years, and 56% women) utilized the GLIM criteria to diagnose malnutrition [41]. Here, the applied cut-off values for interleukin-6, a known pro-inflammatory cytokine, were set within the normal range typically observed in healthy individuals (interleukin-6 > 3.84 pg/mL in males and >2.99 pg/mL in females) [41]. Notably, these threshold values align with those published in other studies targeting community-dwelling older adults [42]. Moreover, another prospective cohort study conducted among Chinese community-dwelling subjects with a median age of 72 years employed high-sensitivity CRP as a marker for inflammation. Notably, the cut-off values were set at ≥0.32 mg/dL for males and ≥0.38 mg/dL for females in order to diagnose malnutrition in accordance with the GLIM guidelines [43]. 

Therefore, given the variety of inflammation thresholds employed in past research, a critical question emerges: What degree of inflammation serves as an accurate indicator of reduced food intake and an elevated risk of malnutrition? This pivotal query allows for the establishment of an exact cut-off point for inflammation, which can be used as a reference point in both clinical and research settings in order to prevent the over- or under-estimate of malnutrition. Furthermore, in clinical practice, the inflammation criterion is often easier and more straightforward to assess compared to reduced food intake, thus making the utilization of the GLIM diagnostic framework more reliable. Therefore, understanding the specific degree of inflammation facilitates a deeper comprehension of the underlying dynamics between inflammation, dietary intake, and overall nutritional status and provides an evidence-based threshold that can help in early identification and risk assessment, which is crucial for timely interventions and the management of malnutrition in geriatric patients.

During our research, we observed a prominent dose–effect relationship between CRP levels and the frequency of moderate to severe reductions in food intake among older hospitalized patients, as measured by the respective item of the MNA-SF. Significantly, inflammation emerged as the most substantial risk factor for both severe and moderate to severe decreases in food intake and malnutrition in this older patient group. It had a more pronounced impact compared to other evaluated risk factors. Importantly, this relationship between inflammation, reduced food intake, and malnutrition remained consistent irrespective of the presence of infection or other comorbidities. It is worth noting that, at a CRP level ranging from 3.0 to 4.99 mg/dL, 19% of patients were found to have a severe decrease in food intake, and 66% experienced a moderate to severe reduction. These proportions were observed to escalate even further with increasing CRP values, further underscoring the dose-dependent relationship between systemic inflammation and nutritional status.

Our data demonstrate a continuous increase in the percentage of patients with both a severe and moderate to severe decrease in food intake at higher levels of CRP. However, inflammation in geriatric hospital patients is a mixture of both chronic and acute inflammation, and this cannot be differentiated by our data. Thus, in pure chronic inflammation, even a lower degree of inflammation may lead to a relevantly reduced food intake, especially over a prolonged period of time. Accordingly, our results suggest that a CRP level of 3.0 mg/dL and above is a good indication of the impact of inflammation on food intake among geriatric patients. 

Several potential limitations of our study warrant discussion. Firstly, the measurement of food intake relied on the food intake component of the MNA-SF, meaning we lacked an objective measurement of food intake and dietary requirements. Additionally, the food intake data refer to the preceding three months; thus, we did not possess specific details regarding the exact duration of diminished food intake and the current food intake status. Secondly, the observed reduction in food intake among geriatric patients was seemingly influenced by multiple risk factors, so the possibility of unaccounted confounding factors persists. Thirdly, our study was not designed to differentiate between acute and chronic states of inflammation. As such, future research should strive to investigate this distinction, particularly in this population, where disease-associated inflammation could substantially contribute to malnutrition. 

## 5. Conclusions

A CRP level of 3.0 mg/dL or above has been associated with diminished food intake in the preceding three months in approximately two-thirds of hospitalized geriatric patients. Such reduced dietary intake can significantly affect nutritional status and is therefore considered an indicator of a high risk of malnutrition in this population.

## Figures and Tables

**Table 1:** Characteristics of the study population.

	Total Population (*n* = 1650)
Gender	
Female (*n*; %)	1047 (63)
Male (*n*; %)	603 (37)
Age (y)	79.6 ± 7.4
Height (m)	1.60 ± 0.10
Actual body weight (kg)	70.8 ± 17.6
BMI (kg/m^2^)	27.3 ± 6.1
MNA-SF, Median (IQR)	10 (7–12)
Malnourished (*n*; %)	457 (28)
At risk of malnutrition (*n*; %)	675 (41)
Normal nutritional status (*n*; %)	518 (31)
MNA-SF, reduction in food intake	
Severe decrease in food intake (*n*; %)	200 (12)
Moderate decrease in food intake (*n*; %)	578 (35)
No decrease in food intake (*n*; %)	872 (53)
CRP (mg/dL)	2.6 ± 5.3
No inflammation (0.0–0.49 (mg/dL), *n*; %)	881 (53)
Mild inflammation (0.5–3.0 (mg/dL), *n*; %)	393 (24)
Moderate to severe inflammation (≥3.0 (mg/dL), *n*; %)	376 (23)

BMI, body mass index; MNA-SF, Mini Nutritional Assessment Short Form; CRP, C-reactive protein. Values are given as mean ± SD, number (%) or median (IQR, interquartile range). Height, body weight, and BMI were measured for 1224, 1337, and 1217 patients, respectively.

**Table 2:** Regression analysis of risk factors associated with moderate and severe decrease in food intake and malnutrition.

	Severe Decrease in Food Intake				
	B	Std. Error	Beta	t	*p* Value
CRP levels (<3 and ≥3 mg/dL)	−0.172	0.021	−0.229	−8.217	**<0.000**
Age	−0.001	0.001	−0.031	−1.123	0.262
Gender	−0.045	0.017	−0.073	−2.693	**0.007**
CCI	0.006	0.004	0.041	1.506	0.511
	Moderate and severe decrease in food intake				
CRP levels (<3 and ≥3 mg/dL)	−0.359	0.034	−0.287	−10.674	**<0.000**
Age	−0.005	0.002	−0.072	−2.709	**0.007**
Gender	−0.198	0.027	−0.194	−7.452	**<0.000**
CCI	0.013	0.007	−0.051	−1.954	0.053
	Malnutrition				
CRP levels (<3 and ≥3 mg/dL)	−0.222	0.029	−0.212	−7.730	**<0.000**
Age	−0.009	0.002	−0.164	−4.993	**<0.000**
Gender	−0.055	0.023	−0.065	−2.442	**0.015**
CCI	−0.015	0.006	−0.067	−2.507	**0.012**

CRP, C-reactive protein; CCI, Charlson Comorbidity Index. Significant *p* values are indicated in bold print. The Charlson Comorbidity Index was used for 1302 patients.

## Data Availability

Additional data are available from the corresponding author upon reasonable request.
